# Supplementary material for: The genome sequence of a cased caddisfly, Molanna angustata Curtis, 1834
Source: Wellcome Open Res. 2024 Jun 13;9:315. [Version 1] doi: 10.12688/wellcomeopenres.22271.1 (PMC11502996; doi:10.12688/wellcomeopenres.22271.1)
Supplement: Peer review history

# Reviewer report: Reviewer response for version 1

**DOI:** 10.21956/wellcomeopenres.24548.r93236

**Published:** 2024-10-24

**Authors:** Halil Ibrahimi

**Affiliations:** 1 University of Prishtina, Pristina, Kosovo (Serbia and Montenegro)

The authors present in this note genome sequencing or *Molanna angustata*. The note is well prepared and represents a great contribution in the field of genome sequencing of caddisflies and as a such it merits to be published. Genome sequencing of aquatic insects is becoming a highly important and necessary tool in understanding patterns of aquatic biodiversity.

Are sufficient details of methods and materials provided to allow replication by others?

Yes

Is the rationale for creating the dataset(s) clearly described?

Yes

Are the datasets clearly presented in a useable and accessible format?

Yes

Are the protocols appropriate and is the work technically sound?

Yes

Reviewer Expertise:

Aquatic insect biodiversity; molecular analysis of aquatic insects

I confirm that I have read this submission and believe that I have an appropriate level of expertise to confirm that it is of an acceptable scientific standard.

# Reviewer report: Reviewer response for version 1

**DOI:** 10.21956/wellcomeopenres.24548.r100365

**Published:** 2024-10-15

**Authors:** Paul B Frandsen

**Affiliations:** 1 Brigham Young University, Provo, Utah, USA

I enjoyed reading this paper on the genome of *Molanna angustata*. It's a nice addition to the growing collection of caddisfly genomes being generated by the Darwin Tree of Life Project. I think the paper has sufficient detail for the analysis to be repeated. I especially appreciated the description of the quality control pipeline, which I think all genome releases should adhere to! The only feedback that I would give is that it would be nice if a photo or illustration of the case could be included. It has quite a unique case, and, for readers, it would be helpful to have a visual.

Are sufficient details of methods and materials provided to allow replication by others?

Yes

Is the rationale for creating the dataset(s) clearly described?

Yes

Are the datasets clearly presented in a useable and accessible format?

Yes

Are the protocols appropriate and is the work technically sound?

Yes

Reviewer Expertise:

caddisfly genomics

I confirm that I have read this submission and believe that I have an appropriate level of expertise to confirm that it is of an acceptable scientific standard.
